# Multiple variations of the urogenital vascular system in a single cadaver: a case report

**DOI:** 10.1186/1757-1626-1-344

**Published:** 2008-11-22

**Authors:** Raghu Jetti, PS Jevoor, Venkata Ramana Vollala, Bhagath Kumar Potu, MV Ravishankar, RD Virupaxi

**Affiliations:** 1 Department of Anatomy, Melaka Manipal Medical College, Manipal University, Manipal, Karnataka, India; 2 Department of Anatomy, Kasturba Medical College, Manipal University, Manipal, Karnataka, India; 3 Department of Anatomy, J.N Medical College, K L E University, Karnataka, India

## Abstract

**Introduction:**

The knowledge of renal vascular anatomy and its variations are very much essential in case of renal transplantation, renal surgeries, uroradiology, gonadal color doppler imaging, in surgeries of aneurysm of abdominal aorta, in gonadal surgeries.

**Case presentation:**

We report a rare combination of vascular variations in a 48 years old male cadaver, included the retroaortic left renal vein opening in to the left common iliac vein, an accessory renal artery arising from the left common iliac artery which supplied the lower end of left kidney and left testicular artery originating from left renal artery. Clinical and embryological back ground of the above mentioned variations have been discussed in this paper.

**Conclusion:**

A deeper understanding of the urogenital vascular variations and their special relations to adjacent vessels is especially significant in avoiding the complications in clinical operation and examination and in recognizing the causes of urinary and genital disorders.

## Introduction

Renal veins are the large veins which lie in front of the renal arteries and drain in to the inferior vena cava at right angles. The left renal vein is three times longer than the right, crosses the posterior abdominal wall and passes in front of aorta to reach the inferior vena cava. Small veins emerge from the testis and unite to form the pampiniform plexus in the spermatic cord, at the superficial inguinal ring four veins arise from the plexus and ascend anterior to the ductus deferens, traverse the inguinal canal, leave the deep inguinal ring as two veins. These veins unite and form a single testicular vein on the posterior abdominal wall and ascend to open in to the inferior vena cava on right side. Where as the testicular arteries are paired vessels that normally originate from the abdominal aorta at the level of second lumbar vertebrae [1].

The location and anatomy of the abdominal vessels is of importance for vascular surgeons, general surgeons, traumatologists, urologists, and radiologists during surgical procedures and for diagnostic reasons. In order to attain a correct diagnosis during radiologic procedures.

and to perform complication-free surgery, knowledge of the anatomy and the congenital anomalies of these vessels is important. In this paper, we report multiple variations involved the retroaortic left renal vein (RLRV) opening in to the left common iliac vein, an accessory renal artery arising from the left common iliac artery and left testicular artery originating from left renal artery. The embryological and clinical significance of the above variations have been highlighted in this communication.

## Case presentation

**Figure 1:**
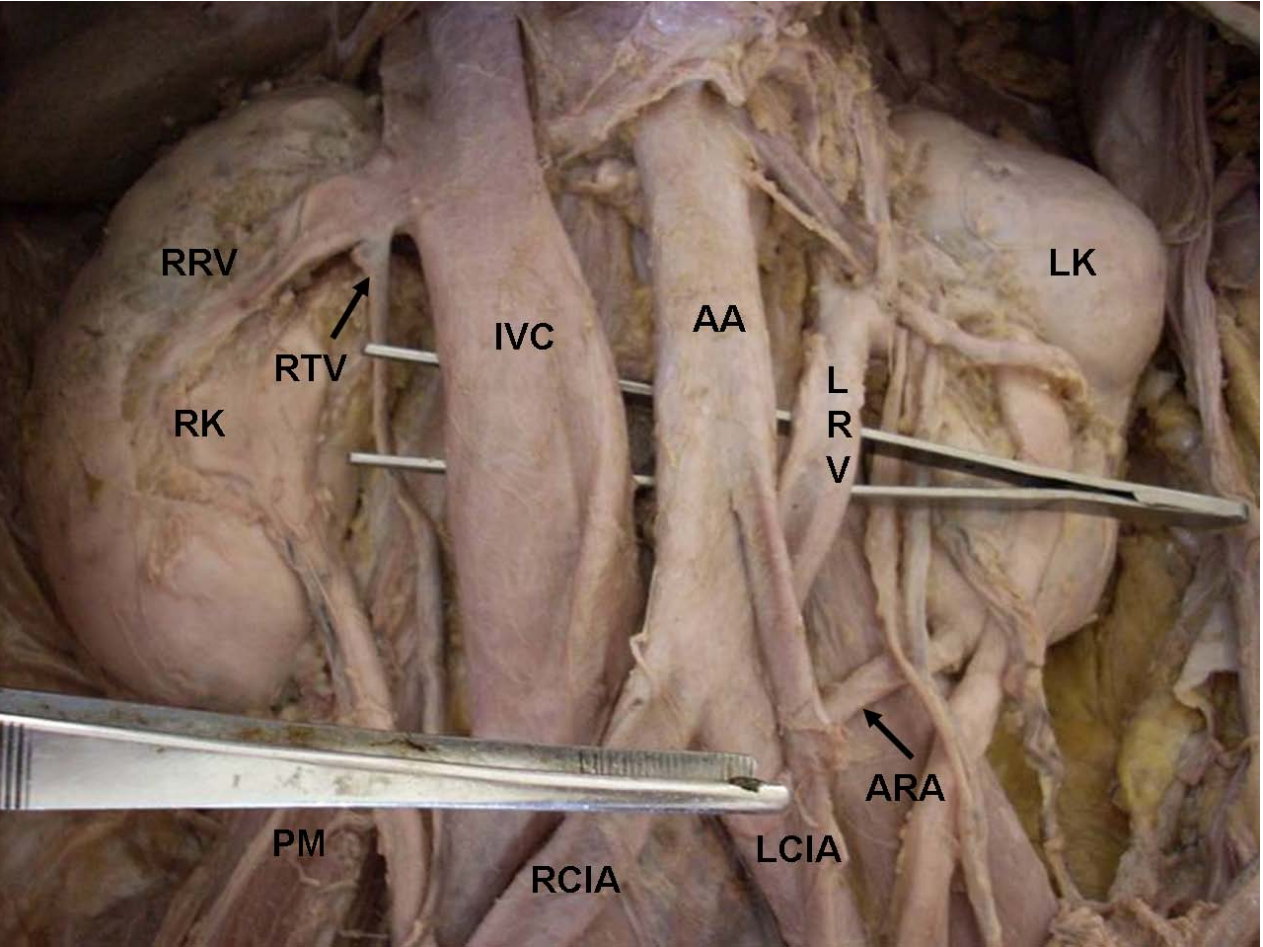
**Showing the abnormal drainage of retro aortic left renal vein into left common iliac vein.** We can also see the abnormal termination of the right testicular vein into right renal vein and origin of accessory renal artery from the left common iliac artery.

**Figure 2:**
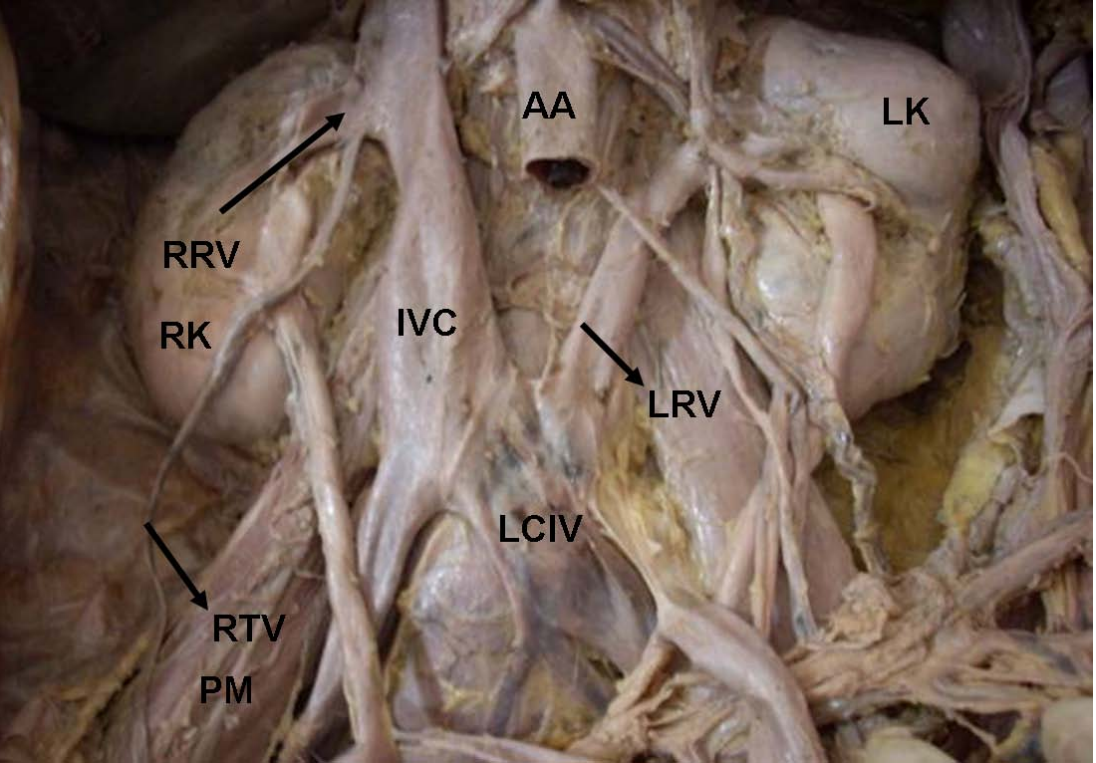
**Showing the abnormal drainage of retro aortic left renal vein into left common iliac vein.** The abdominal aorta is dissected in this figure to show the termination of retro aortic left renal vein.

During routine cadaveric dissection in a 48 years old male cadaver we found the following variations. The left renal vein was 8.5 cm in length and seen coming from the hilum of left kidney below the renal artery and posterior to the pelvis of the left ureter. Near the hilum the vein received left supra renal vein and left testicular vein. Then it passed downwards and medially crossing the psoas major, passed behind the aorta, left common iliac artery and terminated by draining in to the left common iliac vein (Figure 1 &2). The course of the renal arteries was normal but the left renal artery gave origin to the left testicular artery which was 21.3 cms (Figure 1). The lower pole of the left kidney was also supplied by an accessory artery arising from left common iliac artery, which passed upwards and laterally behind the inferior mesenteric artery to reach the left kidney (Figure 1). The length of accessory renal artery was 5.2 cm. All others vessels in the abdominal cavity were normal.

## Discussion

Renal vein anomalies, although usually overlooked and disregarded, have been reported as frequent congenital anomalies [2]. The retroaortic left renal vein anomaly in our case is similar to the type I1 retroaortic left renal vein of Hoeltl et al.,1990, [3] but in our case the vessel terminated into the left common iliac vein instead of the inferior vena cava. This is of particular interest because this kind of anomaly of the left renal vein has not been reported in association with other variations described in this paper.

Haematuria, pain, thrombosis, left renal vein hypertension, and varicocele are some of the reported clinical entities related to RLRV [4]. Furthermore, RLRV poses potential hazards to the surgeon during abdominal aortic surgery. In repair of an abdominal aortic aneurysm where the aorta is mobilized, the RLRV becomes an even larger obstacle. The risk of accidentally damaging a RLRV is higher for the surgeons who routinely dissect the aorta circumferentially to apply the proximal cross clamp. Careful preoperative evaluations are important for establishing the presence of an associated venous anomaly to ensure the success of abdominal aortic surgery [5-7].

The persistence of the left sub-supracardinal anastomoses and the left supracardinal vein, and obliteration of the intersubcardinal anastomoses during embryogenesis might lead to termination of left renal vein into left common iliac vein presented in this case. Many authors have concluded in their study that the 5–6% of individuals, the testicular artery arose from the main or accessory renal arteries [8-13].

Anomalies of the arteries of the renal pedicle carry the risk of serious hemorrhage when using endourological techniques in the treatment of disorders of the pelviureteral junction. Arteries in front of or behind the renal pelvis are the cause of ureteropelvic function obstruction in 15–52% of cases [13]. According to Ravery [9], the explanation of this vascular variation is an embryological one and results from the double movement: the ascend of the kidney and the descend of the testis; the gonadal arteries posses a mesonephrotic origin, so within the descend of the testis, it will be supplied successively from different lower levels, while the upper branches suffer a major atrophy. During the anterior crossing of the kidney, the testis receives two pedicles: a sub renal one and a suprarenal one. The second one will become atrophied and, if persists, will give birth to the arterial variation described in this case.

The accessory renal arteries are seen frequently [14,15]. They enter the kidney either above or below the hilum. Their relations with the nearby structures can vary [16]. Bayramoglu et al. reported bilateral additional renal arteries originating from the abdominal aorta and an additional right renal vein accompanying the additional right renal artery [17].

These anomalies were associated with unrotated kidneys with extrarenal calyces and pelvis. The abnormalities in the renal arteries are mainly due to the various developmental positions of kidney. The kidneys begin their development in the pelvic cavity and then ascend to their final position in the lumbar region. When the kidneys are situated in the pelvis, they are supplied by the branches of common iliac arteries. While the kidneys ascend to lumbar region, their arterial supply also shifts from common iliac artery to the abdominal aorta [18]. But in the present case, though kidneys were situated in the lumbar region, they were supplied by an accessory renal artery arose from the left common iliac artery. This might be due to the failure in the disappearance of the blood vessels which supplied the kidney when it is situated in the pelvic cavity. It is important to be aware that accessory renal arteries are end arteries; therefore, if an accessory artery is damaged, the part of kidney supplied by it is likely to become ischaemic.

The variations which are reported here, have already been reported as individual cases of variations, but occurrence of variations of the renal, accessory renal, and testicular vessels in the same individual have not been reported till date. A deeper understanding of these variations and their special relations to adjacent vessels is especially significant in avoiding the complications in clinical operation and examination and in recognizing the causes of urinary and genital disorders.

## Abbreviations

RRV: right renal vein; RK: right kidney; RTV: right testicular vein; PM: psoas major; IVC: inferior vena cava; AA: abdominal aorta, LRV: left renal vein; LK: left kidney; RCIA: right common iliac artery; LCIA: left common iliac artery; ARA: accessory renal artery; LCIV: left common iliac vein.

## Consent

Written informed consent was obtained from the subject's relative for publication of this case report.

## Competing interests

The authors declare that they have no competing interests.

## Authors' contributions

PS, RJ and VRV did the literature search and wrote the case report and also obtained written consent. BKP conceived the study and helped to draft the manuscript. RS and V helped in the literature search. All authors had gone through the final manuscript and approved it.

## References

[B1] (1995). Gray's anatomy.

[B2] Davis CJ, Lundberg GD (1968). Retroaortic left renal vein. A relatively frequent anomaly. Am J Clin Pathol.

[B3] Hoeltl W, Hruby W, Aharinejad S (1990). Renal vein anatomy and its implication for retroperitoneal surgery. J Urol.

[B4] Arslan H, Etlik O, Ceylan K, Temizoz O, Harman M, Kavan M (2005). Incidence of retro-aortic left renal vein and its relationship with varicocele. Eur Radiol.

[B5] Satyapal KS, Kalideen JM, Haffejee AA, Singh B, Robbs JV (1999). Left renal vein variations. Surg Radiol Anat.

[B6] Karkos CD, Bruce IA, Thomson GJ, Lambert ME (2001). Retroaortic left renal vein and its implications in abdominal aortic surgery. Ann Vasc Surg.

[B7] Kudo FA, Nishibe T, Miyazaki K, Flores J, Yasuda K (2003). Left renal vein anomaly associated with abdominal aortic aneurysm surgery: report of a case. Surg Today.

[B8] Merklin RJ, Michels NA (1958). The variant renal and suprarenal blood supply with data on the inferior phrenic, ureteral and gonadal arteries. J Int Coll Surg.

[B9] Ravery V, Cussenot O, Desgrandchamps F, Teillac P, Martin-Bouyer Y, Lassau JP, Le Duc A (1993). Variations in arterial blood supply and the risk of hemorrhage during percutaneous treatment of lesions of the pelviureteral junction obstruction: Report of a case of testicular artery arising from an inferior polar renal artery. Surg Radiol Anat.

[B10] Machnicki A, Grzybiak M (1997). Selected cases of atypical course of renal and gonadal arteries and veins. Folia Morphol (Warsz).

[B11] Asala S, Chaudhary SC, Masumbuko-Kahamba N, Bidmos M (2001). Anatomical variations in the human testicular blood vessels. Ann Anat.

[B12] Bergman RA, Afifi AK, Miyauchi R Illustrated Encyclopedia of HumanAnatomic Variation:Opus II: Cardiovascular system:Arteries: Gonadal (ovarian and spermatic or testicular) arteries. http://www.anatomyatlases.org/AnatomicVariants/Cardiovascular/Text/Arteries/Gonadal.shtml.

[B13] Petru B, Elena S, Dan I, Constantin D (2007). The morphology and the surgical importance of the gonadal arteries originating from the renal artery. Surg Radiol Anat.

[B14] Singh G, Ng YK, Bay BH (1998). Bilateral accessory renal arteries associated with some anomalies of the ovarian arteries – a case study. Clin Anat.

[B15] Satyapal KS, Haffejee AA, Singh B (2001). Additional renal arteries: incidence and morphometry. Surg Radiol Anat.

[B16] Bordei P, Sapte E, Iliescu D (2004). Double renal arteries originating from the aorta. Surg Radiol Anat.

[B17] Bayramoglu A, Demiryurek D, Erbil KM (2003). Bilateral additional renal arteries and an additional right renal vein associated with unrotated kidneys. Saudi Med J.

[B18] Moore KL, Persaud TVN (2002). The Developing Human: Clinically Oriented Embryology.

